# Measuring health-relevant businesses over 21 years: refining the National Establishment Time-Series (NETS), a dynamic longitudinal data set

**DOI:** 10.1186/s13104-015-1482-4

**Published:** 2015-09-29

**Authors:** Tanya K. Kaufman, Daniel M. Sheehan, Andrew Rundle, Kathryn M. Neckerman, Michael D. M. Bader, Darby Jack, Gina S. Lovasi

**Affiliations:** Department of Epidemiology, Columbia University Mailman School of Public Health, 722 West 168th Street, 8th Floor, New York, NY 10032 USA; Columbia Population Research Center, 1255 Amsterdam Avenue, Room 715, New York, NY 10027 USA; Department of Sociology, Center on Health, Risk and Society, American University, Battelle-Thompkins T-15, 4400 Massachusetts Ave., N.W., Washington DC, 20016 USA; Department of Environmental Health Sciences, Columbia University Mailman School of Public Health, 722 West 168th Street, 11th Floor, New York, NY 10032 USA; NYC Department of Health and Mental Hygiene, Brooklyn District Public Health Office, 485 Throop Avenue, Brooklyn, New York, NY 11221 USA

**Keywords:** Longitudinal data resource, GIS, Public health, Retail environment, Commercial businesses

## Abstract

**Background:**

The densities of food retailers, alcohol outlets, physical activity facilities, and medical facilities have been associated with diet, physical activity, and management of medical conditions. Most of the research, however, has relied on cross-sectional studies. In this paper, we assess methodological issues raised by a data source that is increasingly used to characterize change in the local business environment: the National Establishment Time Series (NETS) dataset.

**Discussion:**

Longitudinal data, such as NETS, offer opportunities to assess how differential access to resources impacts population health, to consider correlations among multiple environmental influences across the life course, and to gain a better understanding of their interactions and cumulative health effects. Longitudinal data also introduce new data management, geoprocessing, and business categorization challenges. Examining geocoding accuracy and categorization over 21 years of data in 23 counties surrounding New York City (NY, USA), we find that health-related business environments change considerably over time. We note that re-geocoding data may improve spatial precision, particularly in early years. Our intent with this paper is to make future public health applications of NETS data more efficient, since the size and complexity of the data can be difficult to exploit fully within its 2-year data-licensing period. Further, standardized approaches to NETS and other “big data” will facilitate the veracity and comparability of results across studies.

**Electronic supplementary material:**

The online version of this article (doi:10.1186/s13104-015-1482-4) contains supplementary material, which is available to authorized users.

## Introduction

Neighborhood investment and use zoning have both been recommended as strategies to promote healthy lifestyles. But these recommendations are almost exclusively based on studies examining neighborhood-health associations at a single point in time rather than changes to the neighborhood environment [1–4]. A stronger evidence base to inform policies that change neighborhood environments requires, at a minimum, studying the health consequences of neighborhood change [5].

While some recent studies include longitudinal data on health behaviors, even in longitudinal studies the neighborhoods are often modeled as static, and health behaviors are considered to be shaped mainly by the concurrent residential environment [6]. As the field moves toward using longitudinal data sources to characterize changing local contexts, there is need to consider both the possibilities and the perils of such measurement over time.

The possibilities have been articulated and are starting to be realized. Longitudinal geographic data have recently become available to characterize dynamic patterns in the distribution and density of health-relevant resources over time [7]. Such longitudinal data offer opportunities to assess how differential access to resources impacts population health, to consider correlations among multiple environmental influences across the life course [8], and to gain a better understanding of their interactions and cumulative health effects [1].

However, little attention has been given to the data management problems inherent to working with longitudinal geographic data. For adequate characterization of how environments change over time, measurement error should be minimized and fairly stable across years or decades. Yet options for ground-truthing (on-site verification) [9] are constrained when working with historical data.

This paper describes methodological issues raised by a data source that has recently been used [10–19] to characterize the commercial environment longitudinally: the National Establishment Time-Series (NETS) database [20]. NETS provides annual establishment-level data for each January beginning in 1990, for US businesses and many nonprofit and government establishments. Our discussion is anchored by our work processing NETS data for the New York–New Jersey–Pennsylvania (NY–NJ–PA) Core Based Statistical Area (CBSA), 1990–2010, a total of 2,815,940 business records. We discuss how changes in spatial accuracy and business classifications in NETS may affect our measurement of trends over time, with approaches to limit bias and check robustness. Our intent is to make future public health applications of NETS data more transparent and efficient, since the size and complexity of the data can be difficult to exploit fully within its two-year data-licensing period.

## Background

The longitudinal NETS database is derived from Dun & Bradstreet’s (D&B) annual register of business information since 1990. D&B’s data collection effort is part of a strategy to develop predictive credit ratings and scores for banking and insurance purposes. Thus businesses have an incentive to report, and D&B has an incentive to maximize accuracy [10, 13]. The merit of D&B and other commercially available business data for neighborhood-effects studies has been previously discussed [21, 22] and the agreement with other methods (both field audits and alternative secondary data sources) has been described [23–27]. Through a partnership with D&B, the longitudinal NETS product was developed by Walls and Associates to capture the dynamics of the US economy [20].

Over 300 NETS variables per business are available to characterize the dynamic business environment, including company name, information on an establishment’s most recent location (city, state, zip code, latitude and longitude), relocation history, industrial classification codes, sales volume, and number of employees. Historical street address information is available for an additional cost, and includes the street address for the establishment’s most recent location, as well as the street address for the origin and destination of every significant move. We purchased this additional data to examine geoprocessing issues. NETS has primarily been used for research on job growth, small businesses, and industry relocation [12–17]. The process of data collection and the organization of NETS data has been described in detail previously in the economic literature [13].

NETS is considered one of the most comprehensive establishment sources available, serving as a census of American businesses for each year since 1990 [13]. Thus, while local environment measures derived from NETS are potentially subject to overcounting, undercounting, and misclassification of businesses, this “big data” resource does not rely on a statistical sampling method but instead is designed to capture all establishments nationally. It would be cost-prohibitive to collect comparable data through field audits, due to the extensive spatial scope of NETS [28]. More crucially, field or even virtual audits [29] conducted in the present cannot capture the environment from multiple time points in the past. NETS data has begun to attract attention from population health researchers, though is not always harnessed for time-varying measures [30–32]. This longitudinal database has potential applications in ecological time-series studies and linkage to human health data.

## Public health applications of NETS

### Ecological studies

**Fig. 1:**
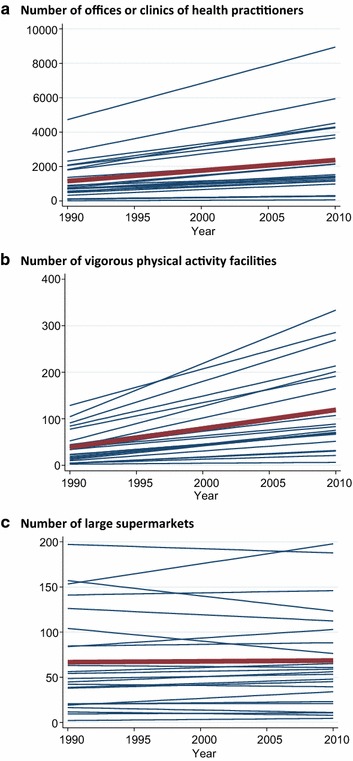
Spaghetti plots of the number of businesses in the specified category, from 1990 to 2010, with each *line* representing one of the 23 counties in the NY–NJ–PA CBSA. *Red line* indicates overall trend

Using an ecological time-series design, NETS data can be used to examine temporal change in the density and distribution of different types of commercial resources. Figure 1 summarizes the variation in the rate of change for a number of businesses in three illustrative categories (offices or clinics of health practitioners, vigorous physical activity facilities, and large supermarkets) across the 23 counties in our CBSA; each line represents the best-fit linear trend for a single county across the 21-year period. For both clinical offices and commercial venues for vigorous physical activity, the county-specific slopes ranged from fairly stable to more than doubling across the two decades. For large supermarkets, in contrast, the average slope was fairly flat and both increasing and decreasing slopes were observed for particular counties. Because NETS data provide geocodes for each establishment, similar analyses can be conducted at multiple geographic scales that can be as small as the street level. Examining relationships with demographic change can reveal emerging or vanishing disparities in the availability of resources by population sociodemographic (income, race, and ethnicity) and geographic characteristics (urban versus rural areas) [7, 11, 33]. Heterogeneity in the degree to which local business environment changes track with other trends, such as changing neighborhood socioeconomic indicators over time, may help to disentangle competing hypotheses to explain persisting disparities in resource access and health behaviors [34–36].

**Fig. 2:**
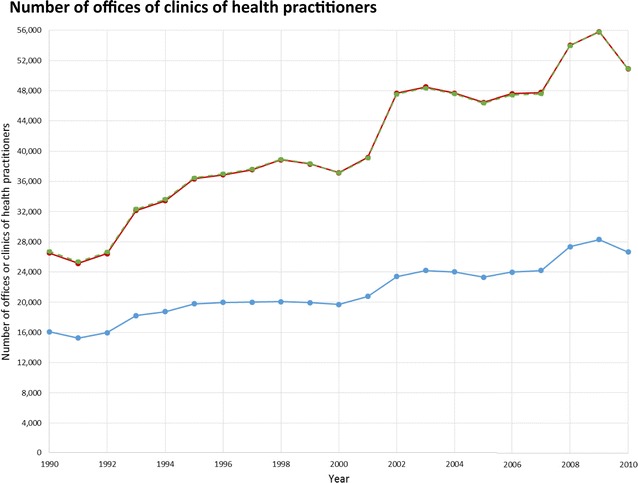
Total number of offices of clinics of health practitioners in NY–NJ–PA CBSA 23 counties, for the years 1990–2010, and the effect of treatment of business categorization and potentially duplicative businesses.  Overall category assignment,  yearly category assignment,  overall category assignment, after collapsing potentially duplicative businesses

**Fig. 3:**
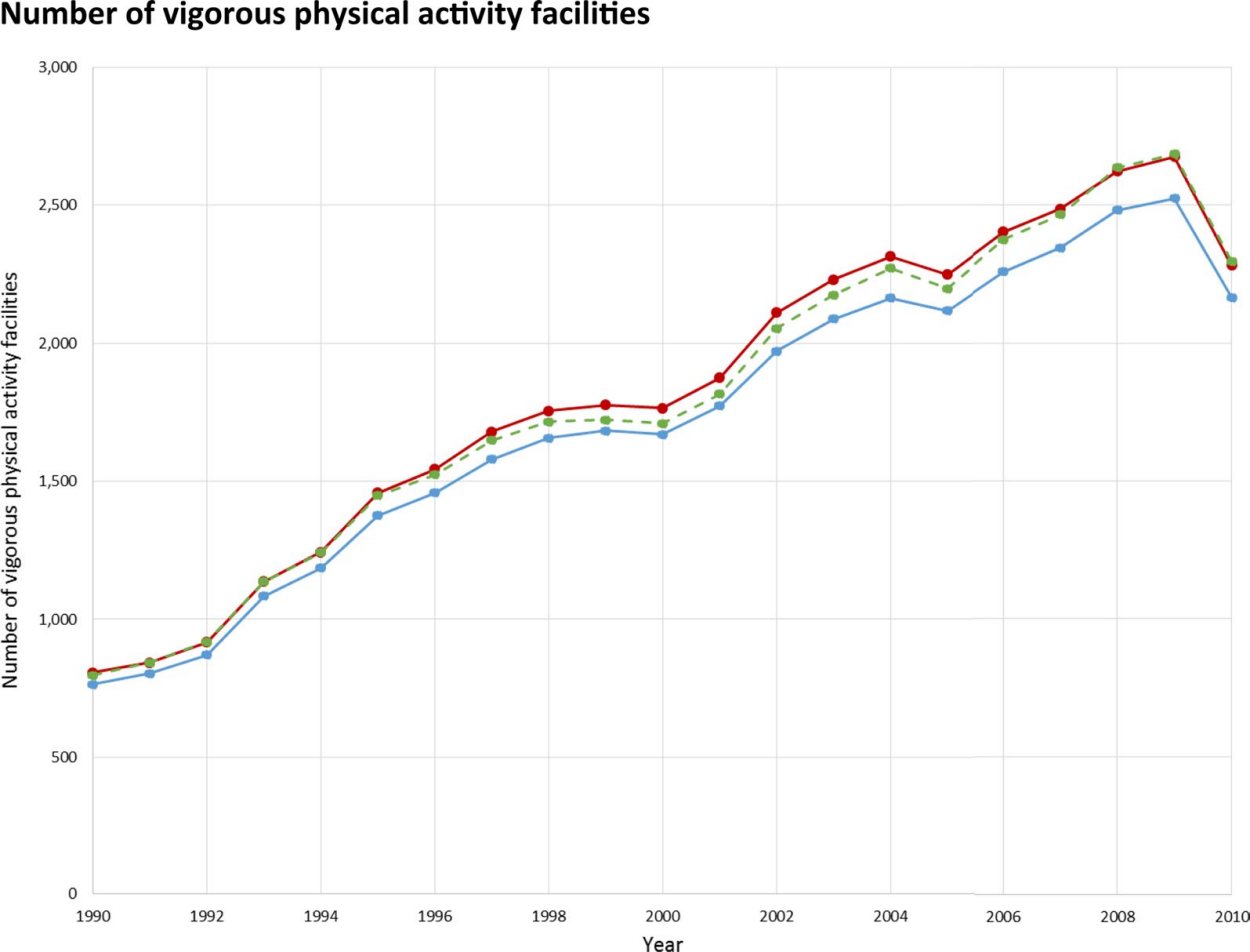
Total number of vigorous physical activity facilities in NY–NJ–PA CBSA 23 counties, for the years 1990–2010, and the effect of treatment of business categorization and potentially duplicative businesses.  Overall category assignment,  yearly category assignment,  overall category assignment, after collapsing potentially duplicative businesses

**Fig. 4:**
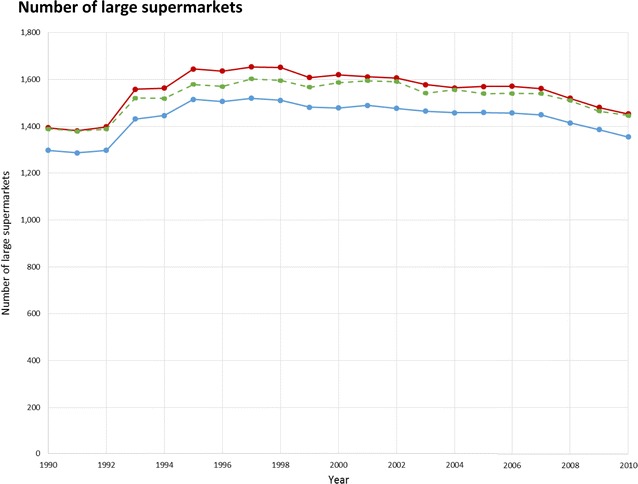
Total number of large supermarkets in NY–NJ–PA CBSA 23 counties, for the years 1990–2010, and the effect of treatment of business categorization and potentially duplicative businesses.  Overall category assignment,  yearly category assignment,  overall category assignment, after collapsing potentially duplicative businesses

Promising strategies to promote health and prevent chronic conditions include creating new opportunities for physical activity and increasing availability of places to buy healthy foods. Likewise, policies restricting access to resources, such as fast food [37] and alcohol [38], have been considered. While the plots in Fig. 1 assume a simple linear trend over time, more complex temporal trajectories (Figs. 2, 3, 4) or discontinuities may also be of interest, particularly for evaluation of policies shaping the business environment. For example, in New York City (NYC) the Food Retail Expansion to Support Health (FRESH) program provides zoning and financial incentives to promote the establishment of neighborhood grocery stores in underserved communities [39]. Using NETS, one can trace the preceding decades for establishment and retention of new grocery stores and compare to rates of change in supermarket density after program implementation.

### Epidemiologic analyses linking industry data to health outcome data

Upon spatially linking NETS with an appropriate health dataset, one can explore associations between changes to the local business environment and ensuing changes in a health outcome of interest. For example, NETS has been used to explore how changes in the food environment affect body mass index of children [10, 11]. Additionally, NETS has been used to create time-varying measures of the number of social destinations and walking destinations to investigate the association of neighborhood built environment with objectively measured body mass index and waist circumference among adults [19], and with walking for transportation [18]. When combined with subjects’ residential history, NETS offers an opportunity to measure contexts experienced over the life course, and may be used to evaluate whether sensitive periods or cumulative exposures in the past two decades have shaped later health.

To expand on the FRESH initiative example above, by pairing NETS with an appropriate health dataset it would also be possible to assess whether increasing the presence of grocery stores in policy-affected neighborhoods is followed by anticipated dietary changes and health benefits [39]. With repeated measures of both environmental exposure data and health outcome data over time, such natural experiments could serve to better establish temporality and causality [40], and to investigate lag times between environmental changes and their impact on health. Use of NETS may allow opportunistic evaluation of multiple natural experiments, thus less subject to timing and fidelity problems encountered when natural experiments focus on a single change, such as one specific supermarket opening.

## Methods for data management, geoprocessing, and business categorization

To implement the research strategies reviewed above, investigators must resolve several data management, geoprocessing, and business categorization issues. While commercially available business data has been used in the past by public health researchers [41–44], and approaches for classifying specific industries have been described in detail [11, 45, 46], the longitudinal nature of NETS introduces new challenges. We explore the stability of spatial accuracy and business classifications over time, as these may affect trends. We illustrate decision points and potential approaches to data cleaning established during our work with the large-scale NETS data for the NY–NJ–PA CBSA. Standardized approaches to using NETS data will facilitate the comparison of results across studies.

### NETS data structure and delivery format

NETS data can be licensed nationally, by state, CBSA, combined statistical area, or other selection criteria to meet specific research needs [20], with pricing dependent on the number of establishments per delivery. Our license for the NY–NJ–PA CBSA comprised business establishments that were ever located in our 23 county region between 1990 and 2010, and the data arrived as 13 tab-delimited American Standard Code for Information Interchange (ASCII) format files that required additional processing to create a true relational database. The datasets varied in structure (wide versus long format), and were partially overlapping. Our data processing and analyses of NETS were generated using StataSE 12.0 (StataCorp, College Station, TX, USA) and SAS 9.4 (SAS Institute Inc., Cary, NC, USA). Code is available from authors upon request. Due to the size of the NETS database, there is a non-trivial run-time for commands in statistical programs on a standard desktop computer.

A nine-digit unique Data Universal Numbering System (DUNS) number, assigned by D&B to identify and track each business establishment, was used to merge datasets. Our efforts to synchronize data processing with a project using NETS in King County, Washington revealed that file naming may vary across data distributions from Walls and Associates, so we note file content where necessary, rather than relying exclusively on the file name as delivered to us.

### Creating a comprehensive address file

Location information is contained in several of the dataset files. The most recent address is provided, along with the most recent company name, in a wide format dataset with one observation for each business establishment. The first address recorded for each establishment is in the address first file. For businesses that relocate there is a long format (multiple observations per establishment) data file that contains the origin and destination addresses for each significant move (a move in which both the five-digit zip code and street address changed between years). Geographical coordinates in NETS are provided by D&B for the most recent address, and for the origin and destination addresses of each significant move. For the 2,815,940 businesses in our data delivery, 10 % of the businesses had at least one significant move, with businesses relocating up to eight times between 1990 and 2010. The level of geocoding accuracy for each set of coordinates is noted in NETS as block face, street segment, block group, census tract centroid, or zip code.

A business with no significant moves will have two addresses on file in NETS: the first reported address and the most recent address, which were expected to be equivalent. For an establishment that moved once, the most recent address was expected to be equivalent to the destination from the most recent move, and the origin of that single move should be the same as the first reported address. However, the same address may be recorded with deviations in the character string, and therefore geocode to a different location. To assign a single location to each business for each year while preserving as much information as possible, we manipulated the address files such that for a given location we retained two versions of the address string (for illustration see Additional file 1).

### Geocoding business locations

Using the addresses for each location, we attempted to re-geocode all 3,161,715 business locations in our comprehensive address file, and compared match rates and levels of geocode accuracy over time. Due principally to significant moves and the inclusion of headquarter offices, we received and subsequently excluded data on business locations (3 % of those received) that were geocoded to be outside of our 23 county region.

Three geocoding services were used to geocode each address. First, we used Geosupport (Desktop Edition) 10a, a customized tool developed by the NYC Department of City Planning to accurately match parcel-based NYC addresses. Geosupport includes street name aliases and corner lots with alternative addresses, and is the gold-standard geocoding software for NYC. We additionally used two services incorporated into ESRI 2010 Business Analyst (ESRI, Redlands, CA, USA) to match point location (rooftop or tax parcel-based centroid) or street segment (range-interpolated). A priori we chose to prioritize address coordinates returned by Geosupport over the ESRI 2010 Business Analyst point level results, which both took priority over the less spatially precise Business Analyst street segment results. For 37,670 (1.3 %) businesses, addresses failed to geocode with any of our three geocoders, in which case we used the longitude and latitude provided with the data delivery.

**Fig. 5:**
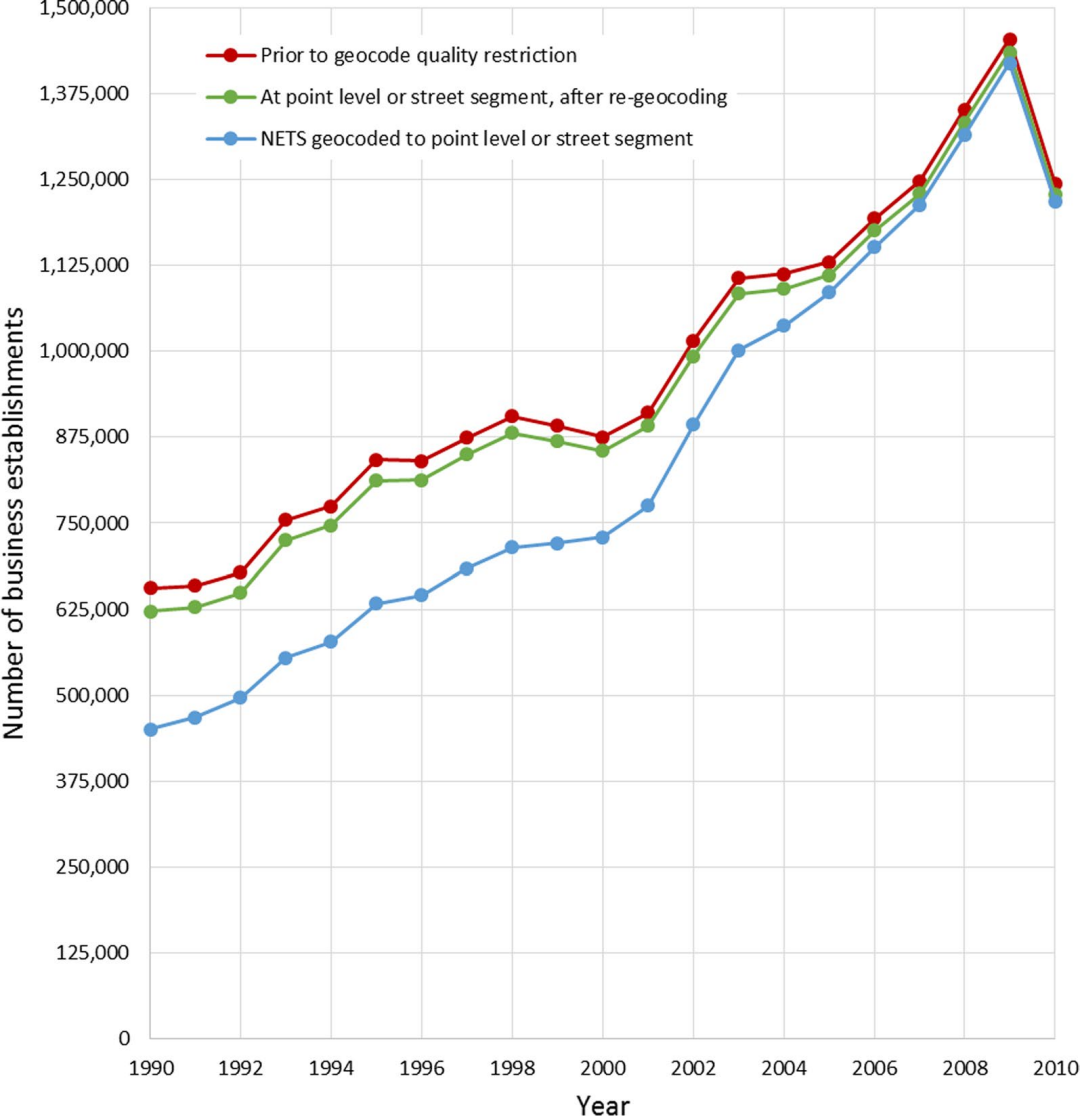
Total number of business establishments in NY–NJ–PA CBSA 23 counties, for the years 1990–2010, and the effect of geoprocessing decisions

NETS documentation suggested that because D&B only began geocoding their establishment data in 2000, geocodes for prior years tend to be less accurate [47]. Walls and Associates seek to assign establishments in the same building the same geocoded coordinates and to ensure consistency since D&B has changed geocoding vendors [47]. As delivered, 31 % of businesses in NETS had not been geocoded with precision better than the zip code level in 1990, 16 % in 2000, and 2 % in 2010 (Fig. 5). After re-geocoding, 5 % of businesses in 1990 still did not have geocodes with precision better than the zip code level, 2 % in 2000, and 1 % in 2010. An additional table shows this in more detail (see Additional file 2). Following re-geocoding, we restricted attention to business locations with a final prioritized address coordinate geocoded to the point level or street segment. Our resulting analytical sample consisted of 2,962,119 business locations (2,701,356 businesses) in our 23 county region with geocode accuracy of street level or higher.

Because address strings for the same location can vary greatly and because our three geocoders return matched addresses in different formats, the final prioritized decimal degrees coordinates were projected to Universal Transverse Mercator North American Datum 1983 zone 18 North and rounded to the nearest 10 m. Local projected coordinate systems ensure accurate distance calculations.

### Identifying businesses of interest

Businesses considered health-relevant and pertinent to current research interests were categorized into one of 25 mutually exclusive researcher-defined categories, representing the food environment, medical facilities, alcohol outlets, physical activity facilities, and other destinations of daily living (Table 1). The development of these category definitions has been iterative, and will likely continue as we pursue focused research questions. We describe elements of the process with relevance to these or future category definitions.

**Table 1:** Total number of businesses in each of the 25 researcher-defined categories, 1990–2010, in NY–NJ–PA CBSA 23 counties

	n	%
Bar or other public drinking place	10,726	0.40
Liquor store	7872	0.29
Fish market	1487	0.06
Fruit and vegetable market	3089	0.11
Natural food market or nut store	3541	0.13
Meat market	2986	0.11
Large supermarket	2778	0.10
Convenience store or small grocery store	40,336	1.49
Fast food restaurant	5714	0.21
Pizza restaurant	7805	0.29
Other restaurant—not fast food, not pizza	54,344	2.01
Bakery or candy/confectionary store	12,022	0.45
Warehouse or discount department store selling food	79	0.00
Multi-use physical activity venue	7055	0.26
Light/moderate physical activity venue	6965	0.26
Vigorous physical activity venue	4589	0.17
Urgent care or hospital facility	2358	0.09
Office or clinic of health practitioner	98,793	3.66
Residential facility with health care	2725	0.10
Pharmacy	9593	0.36
Mental health care	10,392	0.38
Dental care	23,584	0.87
Bank	8056	0.30
Credit union	1019	0.04
Other potential destination	214,052	7.92
Categorized businesses	541,960	20.06
Not categorized	2,159,396	79.94
Total businesses	2,701,356	

An eight-digit Standard Industrial Classification (SIC) code and, where applicable, the most recently reported company name, trade name, sales volume, or employee count, were used to assign businesses to one researcher-defined category. Definitions were applied uniformly across all years (definitions provided in Additional file 3). Many of the definitions we used to categorize businesses were based on prior work using D&B data [45], and informed by existing literature using commercial databases to identify food sources such as restaurants and supermarkets [25, 48, 49], and physical activity facilities such as private gyms [50–55]. Additional potentially relevant SIC codes were identified for consideration from the list of over 18,750 eight-digit SICs from which establishments choose, or D&B assigns, an SIC code to designate the type of business based on the establishment’s primary activity.

For each SIC that seemed relevant to a category definition but was not selected based on previous publications, we examined a random sample of businesses reporting that SIC in the year 2010. If there were fewer than fifty establishments for a given SIC code in 2010, all of those businesses were reviewed. A random sample of 20 % of the businesses was reviewed if the frequency of the SIC in 2010 was 50–250. We reviewed a random sample of 50 businesses for SIC with a frequency over 250 in 2010. We selected 2010 since more recent businesses were more likely to have an active website or valid phone number for further review of relevancy. The company and trade name, as well as additional online information if available, were used to evaluate the relevance of the SIC to our categories of interest. If necessary, we verified by telephone. An SIC was included in one of our definitions if at least 50 % of the random sample of businesses represented an establishment relevant to that category. Some of our definitions included text searches of the company and trade name. For example, a name-based search was applied for Young Men’s Christian Associations (YMCA) as well as for Jewish Community Centers (JCC), because their gym facilities are not consistently captured by a given SIC due to the range of services offered. However, businesses are included in NETS under many synonyms and spelling variations of the company and trade name, which makes them challenging to find. Reviewing an alphabetical list of company and trade names in our NETS database informed our search terms.

Importantly, identification of businesses that were relevant to each category required clear conceptualization. For example, we defined commercial physical activity facilities as venues for physical activity likely to be marketed to local residents. As such, an SIC was generally included in our definition if it was a facility, building, court, field, rink, or other structure, that was potentially accessible to the public or to members for active participation in physical activity. Many SICs were excluded because more than half of the randomly sampled businesses were organizations, associations, leagues, clubs or other businesses that, though associated with opportunities for physical activity, did not represent a location at which to be physically active. SICs were also excluded if the businesses were not regularly accessible to local residents (e.g., seasonal camps) or because they were spectator locations (e.g., dance performance venues).

Many of our researcher-defined categories were originally conceived as broad groupings of businesses (i.e., medical facilities, food environment, physical activity facilities), which were then subdivided to consider business categories within that could differentially affect health. For example, we categorized each physical activity facility based on the metabolic equivalent (MET) of the physical activity generally offered there, as light/moderate (1.6–5.9 METS) or vigorous (≥6 METS), using the Compendium of Physical Activity [56]. Where available, we used MET values that were supported in published literature, rather than estimated. If an activity was not included in the Compendium, a MET value was assigned based on similar activities. We also created a multiuse category to account for the wide variety of physical activity opportunities offered at health clubs and gyms, for which assigning any single MET value would offer an inadequate characterization. These subcategories enable us to compare trends in access to different types of physical activity facilities over time.

### Businesses categorization integrating multiple years of data

A key challenge of categorizing businesses from this longitudinal data source was deciding how to integrate information from multiple years. A single classification across all years was sought to avoid having a business “disappear” and then “reappear” in time trends due to inconsistencies in the SIC code assigned to the business in different years.

Of the 2,701,356 businesses in our final sample, 7.7 % changed primary SICs over time. If a business reported one primary SIC code for at least 75 % of the years it was in operation, that majority SIC code was used to represent the business type across all years. For the 4 % of businesses that did not report a consistent SIC at least 75 % of the time, we considered assigning a single SIC code based on manual review of the company and trade names and all the SICs reported. However, this approach was time-prohibitive in a CBSA as large as ours, with over 20,000 establishments to manually check. A random sample of 200 businesses was manually reviewed to understand the changes and to establish feasible data coding options.

For businesses where an SIC was reported for more than 50 % but less than 75 % of the time, our manual SIC assignment was not commonly in agreement with the most common SIC. Instead, over three-quarters of the manual assignments corresponded to the most recently reported SIC. Inspection revealed a trend toward using more specific SIC codes in recent years; for example, a business with a general code, such as “Educational”, in early years may later switch to a more specific code, such as “Martial Arts”. Thus, the most recently reported primary SIC was used to characterize the overall nature of businesses with inconsistent SIC codes (those whose most frequently reported SIC was reported less than 75 % of the time). By using the most recent SIC to classify businesses, we maximized the chances that any misreporting had been corrected, and we were more likely to specifically capture the primary market of the business. However, we note that many establishments that were manually reviewed to assess changing SICs were judged to be classifiable by multiple SICs. For example, a business offering horseback riding could classify itself as a “riding club, membership”, “horseback riding, general”, “riding stable”, “saddlehorse rental”, “horse farm”, “animal boarding services”, or “training services, horses (except racing horses)”.

For a sensitivity analysis we tested a second method of assigning businesses to researcher-defined categories in which we allowed the category to change year-to-year. Businesses could potentially be assigned to a different category yearly based on the SIC and, where applicable, the company and trade name, sales volume, and employee count specific to each year the establishment was in operation. This alternate method might be particularly relevant for food environment studies, because supermarkets are commonly distinguished from mid-sized and smaller grocery stores not by SIC code but by indicators of store size such as sale volume and number of employees. Visual comparisons showed only small differences in the time trend for businesses per category using the year-to-year changing versus the single overall category assignment (Figs. 2, 3, 4).

### Calculating business counts

Twenty percent of the businesses in our 23 county region fall into one of our 25 researcher-defined health-relevant categories (Table 1). The five most common industries among the uncategorized businesses in our dataset were “Business activities at non-commercial site”, “Business services, not elsewhere classified”, “General practice attorney, lawyer”, “Beauty shops”, and “Real estate brokers and agents”.

Once categorized, NETS can be used to assess the presence or absence of businesses in a particular category for a given geographic area. NETS can further be used to estimate the count or density of such businesses. The uncategorized businesses can also be used when calculating a measure of total retail density, though some caution is warranted given the highly variable size of businesses and the potential for small or online businesses to be associated with a home address or other location not actually frequented by consumers.

In calculating business counts for our researcher-defined categories we encountered extreme high values, which were revealed to include multiple concurrent instances for a given business, with some variation in the name field. We also observed instances where multiple limited liability companies (LLCs) were listed at the same address, which presumably was a base of operations or legal office serving multiple companies. We judged that these co-locations could lead to over-counting the number of businesses in a given category. However, the practice of locating multiple similar businesses in the same building is common for industries such as the offices of health practitioners. To better understand the extent to which our results would depend on our treatment of businesses in the same category that were co-located, we developed a method to collapse businesses to a single record if they were in the same researcher-defined category, in the same year, at the same location.

Collapsing potentially duplicative businesses resulted in a 12–66 % decrease in the number of businesses in a given category in a given year (Figs. 2, 3, 4). Among our researcher-defined categories, offices or clinics of health practitioners were frequently co-located, as were light/moderate physical activity facilities, resulting in a substantial number that could be collapsed. The effective number of businesses may be better captured by the collapsed or raw count depending on the business type and research question, so a sensitivity analysis approach could be considered. The collapsed count may be appropriate when a single record or multiple co-located business records would represent similar levels of service access (e.g., a physical activity facility with multiple instructors sharing a space, who could operate either as part of a single business, or each with their own small business, unbeknownst to customers and local residents). Alternatively, collapsing may not be helpful where multiple business records at a single address represent meaningful variety of related services (e.g., a medical office building, where more distinct businesses may mean that a greater variety of specialized diagnostic and treatment options are available). Businesses in different categories at the same location were always considered distinct, even if located at the same address, to accommodate large commercial buildings or areas (e.g., shopping malls).

## Discussion of limitations and considerations for future research

While longitudinal geographic data sources may be used increasingly over time as a result of information sharing policies and technology, it is necessary to assess whether the quality of the data is consistent over time. Changes in geocoding accuracy and business classifications may affect aggregate measures and trends of the distribution of commercial resources. We re-geocoded and categorized longitudinal data with multiple potential descriptive and etiologic uses in public health. We found that our re-geocoding limited the number of establishment locations with low geocoding precision. In using business microdata like NETS, researchers must determine how to treat the substantial number of co-located businesses with similar or identical SIC codes. As noted above, decisions to collapse co-located businesses can have a substantial impact, reducing business density by as much as 66 %. Aggregate time trends within categories were fairly robust to decisions to allow businesses to change categories year-by-year or to collapse co-located records in the same category, but the effect of such decisions on the count of establishments varied by business category.

Like most secondary datasets, NETS has several limitations. Though it is considered one of the most comprehensive databases of establishments [13], some businesses may not be included. Particularly, NETS is unlikely to capture short-lived businesses, including seasonal businesses, since the underlying D&B data is a cross-sectional snapshot from January of each year. Further, businesses categorized here as health-relevant are not definitive, but rather are presented to illustrate the range of possibilities and issues raised while implementing such categorization. Comparing the added-value or contribution of each component of a definition may be informative for future research [28]. At present, approaches to identify and codify health-relevant resources are inconsistent across studies [57], perhaps accounting for some discrepancies in research findings. We hope to encourage comparability and transparency. The operational definitions of any business category may influence the validity of findings, and methods to develop such definitions should be described along with the definitions themselves.

Researchers have previously questioned the precision of the geocodes that are provided with NETS [58]. The geocoding method affects the validity of spatial epidemiology studies [59]. Thus, much of our effort went towards creating a comprehensive address file with every location accounted for and re-geocoded. Restricting attention to business locations geocoded with precision better than the zip code level would differentially exclude older businesses in the earlier years, particularly if relying only on the original NETS geocodes (Fig. 5). While we were able to improve the spatial accuracy of the NETS data, re-geocoding all business locations in NETS was a large investment of time and resources. Developing code processes and experimenting with file formats for the ArcGIS software package was computationally intensive due to the size of the NETS database (for information in greater detail on our geocoding processes and technology recommendations see Additional file 4). While best practice geocoding requires manually reviewing and re-matching addresses for quality control, extensive data cleaning was not deemed feasible. Geocoding historical address data may be particularly problematic due to street names changing over time; for this reason, it may be appropriate for future projects to explore the multi-staged use of archived geocoders [59]. Though geoprocessing NETS is a large investment, the accuracy of business establishment locations is central to valid aggregate measures of commercial business access.

In addition to positional error, another threat to validity for studies deploying NETS data is misclassification error. A study by Liese et al. [60] suggested that review and reassignment of classification codes can reduce misclassification. By assigning a single SIC to each business if the reported SIC code changed over time, our goal was to reduce error from inconsistent reporting practices, particularly early in the study period. Any change in the number of businesses per category would then be attributable to establishments opening, closing, or moving, rather than variation in a business establishment’s reporting over time.

Our categories are defined primarily with SIC codes because of previous research experience using SICs [45, 46] and the availability of published definitions in the literature [42, 52]. However, NETS also includes North American Industrial Classification System (NAICS) codes and a NAICS-SIC crosswalk. As of 1997, NAICS is the industry classification system adopted by the statistical agencies of the United States, as it reflects changes in the US economy and enables comparable statistics across North America [61]. For exploratory purposes we translated our definitions to NAICS using the provided crosswalk. SIC codes outnumber NAICS codes by approximately 16–1, thus in merely using the crosswalk many of our sub-categories were no longer distinct.

We recognize that there is a limit to the granularity in categorization that can be achieved using SIC codes in NETS, and caution against trying to parse businesses into very fine categories, even if deemed health- or socially-relevant. Likewise, although businesses often target a specific population, such as physical activity opportunities tailored to children, further refinement and tailoring would not always be straight forward based on the variables in NETS. We considered further subcategorization (e.g., subcategorizing physical activity venues into yoga or pilates studios) but this level of specificity was not supported by our checks of SIC codes, and would require a high level of dependence on potentially idiosyncratic text search strategies. We use name-based searches in some of our definitions because relying solely on classification codes to categorize businesses, particularly food outlet types, may result in substantial error [60]. However, text searches may be biased by the investigator’s language and familiarity with particular business naming conventions. Even valid text search strategies for current businesses might not work adequately for decades past; company and trade names change over time, and there may be temporal change in how an industry is defined. For example, as previously [45], we used a list of the top 100 limited-service chain brands [62] in a name-based search to identify fast-food restaurants. This list, however, was from 2006 and may not include national-chain fast-food restaurants from the 1990s or early 2000. To capture all relevant resources, locally tailored or archived business lists should be considered [23].

NETS data have been imputed by Walls and Associates with a focus on longitudinal accuracy, using each additional year to inform the number of employees and total sales in previous years [63]. As a result, there is a time-series effect in which the longer an establishment has been in operation, the more likely missing data will have been imputed by Walls and Associates [64]. At present, our definitions for supermarkets, convenience stores or small grocery stores, and other potential destinations incorporate data on the number of employees or sales in the most recent year of business. We have not distinguished between actual and estimated values when using data on the number of employees or sales to inform categorization, but this distinction is flagged within the data and may be pertinent to future research questions.

## Conclusion

Longitudinal geographic information on the spatial distribution of commercial resources has the potential to highlight multiple contexts that influence population health. NETS enables integration of multiple measures of local businesses in the environment [65] and the examination of the retail ecology in its entirety [66] over time. Further methodological work to characterize the changing access to commercial physical activity facilities, food outlets, medical facilities, and other health-relevant businesses over the past two decades will set the stage for future etiologic investigations using longitudinal data. Despite limitations of NETS, the volume of information available in this unique historical database makes it a valuable data source with promise to improve understanding of how the dynamic retail environment affects population health.

## Additional files

10.1186/s13104-015-1482-4 Sources of address information in NETS used to build comprehensive address file.
10.1186/s13104-015-1482-4 Comparing match rates and geocoding accuracy for business locations in NY–NJ–PA CBSA 23 counties, for the years 1990, 2000, and 2010, prior to quality restricting business locations.
10.1186/s13104-015-1482-4 Definitions for 25 health-relevant researcher-defined categories.
10.1186/s13104-015-1482-4 Information in greater detail on our geocoding processes.

## Abbreviations

NETS: National Establishment Time-Series
NY–NJ–PA: New York–New Jersey–Pennsylvania
CBSA: Core Based Statistical Area
D&B: Dun & Bradstreet
NYC: New York City
FRESH: Food Retail Expansion to Support Health
ASCII: American Standard Code for Information Interchange
DUNS: Data Universal Numbering System
SIC: Standard Industrial Classification code
YMCA: Young Men’s Christian Associations
JCC: Jewish Community Centers
MET: metabolic equivalent
NAICS: North American Industrial Classification System
LLC: limited liability companies
