# Supplementary figures and images for: Measuring health-relevant businesses over 21 years: refining the National Establishment Time-Series (NETS), a dynamic longitudinal data set
Source: BMC Res Notes. 2015 Sep 29;8:507. doi: 10.1186/s13104-015-1482-4 (PMC4588464; doi:10.1186/s13104-015-1482-4)

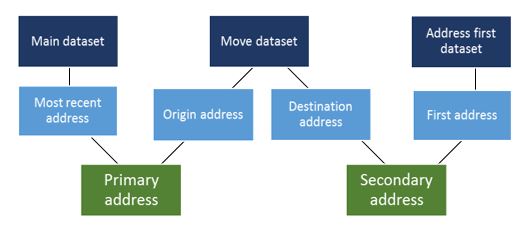


**Source dataset**

**NETS variable**

**Created address variable**

Supplement: Supplementary file 1 — 10.1186/s13104-015-1482-4 Sources of address information in NETS used to build comprehensive address file. [file 13104_2015_1482_MOESM1_ESM.docx]
