# Supplementary material for: Measuring health-relevant businesses over 21 years: refining the National Establishment Time-Series (NETS), a dynamic longitudinal data set
Source: BMC Res Notes. 2015 Sep 29;8:507. doi: 10.1186/s13104-015-1482-4 (PMC4588464; doi:10.1186/s13104-015-1482-4)
Supplement: Supplementary file 2 — 10.1186/s13104-015-1482-4 Comparing match rates and geocoding accuracy for business locations in NY–NJ–PA CBSA 23 counties, for the years 1990, 2000, and 2010, prior to quality restricting business locations. [file 13104_2015_1482_MOESM2_ESM.docx]

1. NETS, as delivered

|  | **1990** | | **2000** | | **2010** | |
| --- | --- | --- | --- | --- | --- | --- |
| **Block Face** | 449,774 | 69% | 728,179 | 83% | 1,215,280 | 98% |
| **Street Segment** | 699 | 0% | 1,381 | 0% | 2,647 | 0% |
| **Block Group** | 1,100 | 0% | 2,040 | 0% | 1,601 | 0% |
| **Census Tract Centroid** | 1,972 | 0% | 3,599 | 0% | 2,367 | 0% |
| **ZIP** | 202,496 | 31% | 140,238 | 16% | 22,587 | 2% |
| **Total** | 656,041 | 100% | 875,437 | 100% | 1,244,482 | 100% |

1. FINAL PRIORITIZED X-Y COORDINATES

|  | **1990** | | **2000** | | **2010** | |
| --- | --- | --- | --- | --- | --- | --- |
| **Block Face/ Point Level** | 557,993 | 85% | 774,089 | 88% | 1,114,997 | 90% |
| **Street Segment** | 64,372 | 10% | 81,244 | 9% | 113,947 | 9% |
| **Block Group** | 198 | 0% | 404 | 0% | 760 | 0% |
| **Census Tract Centroid** | 547 | 0% | 1,059 | 0% | 1,058 | 0% |
| **ZIP** | 32,946 | 5% | 18,641 | 2% | 13,720 | 1% |
| **Total** | 656,056 | 100% | 875,437 | 100% | 1,244,482 | 100% |
