# Supplementary material for: Measuring health-relevant businesses over 21 years: refining the National Establishment Time-Series (NETS), a dynamic longitudinal data set
Source: BMC Res Notes. 2015 Sep 29;8:507. doi: 10.1186/s13104-015-1482-4 (PMC4588464; doi:10.1186/s13104-015-1482-4)
Supplement: Supplementary file 3 — 10.1186/s13104-015-1482-4 Definitions for 25 health-relevant researcher-defined categories. [file 13104_2015_1482_MOESM3_ESM.docx]

| **Business category** | **Definition** (for SAS 9.4) | **SIC description** |
| --- | --- | --- |
| **Bar or other public drinking place** | *Includes 13 SIC codes representing drinking places (5813) and members only bars and restaurants (86410401).*  if SIC in (58130000:58139999,86410401) | \| 58130000 \| Drinking places \| \| --- \| --- \| \| 58130100 \| Bars and lounges \| \| 58130101 \| Bar (drinking places) \| \| 58130102 \| Beer garden (drinking places) \| \| 58130103 \| Cocktail lounge \| \| 58130104 \| Saloon \| \| 58130105 \| Tavern (drinking places) \| \| 58130106 \| Wine bar \| \| 58130200 \| Night clubs \| \| 58130201 \| Cabaret \| \| 58130202 \| Discotheque \| \| 58130203 \| Gentleman's club \| \| 86410401 \| Bars and restaurants, members only \| |
| **Liquor store** | *Includes 6 SIC codes for retail or whole-sale liquor stores (5921), as well as a text search for the word “liquor” in the company name of businesses reporting any of 14 SIC codes for beer, wine, and distilled beverages. (Note: some supermarkets may offer alcohol for sale, but these are not included).*  if SIC in (59200000:59299999)  or (SIC in (51800000:51899999) and (index(CompanyHere,'LIQUOR') > 0)) | \| 59210000 \| Liquor stores \| \| --- \| --- \| \| 59210100 \| Wine and beer \| \| 59210101 \| Beer (packaged) \| \| 59210102 \| Wine \| \| 59219900 \| Liquor stores, nec \| \| 59219901 \| Hard liquor \|  \| 51810000 \| Beer and ale \| \| --- \| --- \| \| 51819900 \| Beer and ale, nec \| \| 51819901 \| Ale \| \| 51819902 \| Beer and other fermented malt liquors \| \| 51819903 \| Porter \| \| 51820000 \| Wine and distilled beverages \| \| 51820100 \| Wine \| \| 51820101 \| Brandy and brandy spirits \| \| 51820102 \| Wine coolers, alcoholic \| \| 51820200 \| Liquor \| \| 51820201 \| Cocktails, alcoholic: premixed \| \| 51820202 \| Neutral spirits \| \| 51829900 \| Wine and distilled beverages, nec \| \| 51829901 \| Bottling wines and liquors \| |
| **Fish market** | *Includes 3 SIC codes for fish and seafood markets (542101).*  if SIC in (54210100:54210199) | \| 54210100 \| Fish and seafood markets \| \| --- \| --- \| \| 54210101 \| Fish markets \| \| 54210102 \| Seafood markets \| |
| **Fruit and vegetable market** | *Includes 4 SIC codes for fruit and vegetable markets (5431).*  if SIC in (54310000:54319999) | \| 54310000 \| Fruit and vegetable markets \| \| --- \| --- \| \| 54319900 \| Fruit and vegetable markets, nec \| \| 54319901 \| Fruit stands or markets \| \| 54319902 \| Vegetable stands or markets \| |
| **Meat market** | *Includes 3 SIC codes for meat markets (542102).*  if SIC in (*54210200: 54210299)* | \| 54210200 \| Meat markets, including freezer provisioners \| \| --- \| --- \| \| 54210201 \| Food and freezer plans, meat \| \| 54210202 \| Freezer provisioners, meat \| |
| **Large supermarket** | *Includes 16 SIC codes identifying grocery stores (5411) for businesses with at least $2 million in annual sales in the most recent year or at least 18 employees in the most recent year.*  if SIC in (54110000:54119999) and ((SalesHere>=2000000 and SalesHere ne .) or (EmpHere>=18 and EmpHere ne .)) | \| 54110000 \| Grocery stores \| \| --- \| --- \| \| 54110100 \| Supermarkets \| \| 54110101 \| Supermarkets, chain \| \| 54110102 \| Supermarkets, greater than 100,000 square feet (hypermarket) \| \| 54110103 \| Supermarkets, independent \| \| 54110104 \| Supermarkets, 55,000 - 65,000 square feet (superstore) \| \| 54110105 \| Supermarkets, 66,000 - 99,000 square feet \| \| 54110200 \| Convenience stores \| \| 54110201 \| Convenience stores, chain \| \| 54110202 \| Convenience stores, independent \| \| 54119900 \| Grocery stores, nec \| \| 54119901 \| Cooperative food stores \| \| 54119902 \| Delicatessen stores \| \| 54119903 \| Frozen food and freezer plans, except meat \| \| 54119904 \| Grocery stores, chain \| \| 54119905 \| Grocery stores, independent \| |
| **Convenience store or small grocery store** | *Includes 3 SIC codes for convenience stores (541102), and includes 16 SIC codes for grocery stores (5411) if they reported fewer than 5 employees in the most recent year.*  if SIC in (54110200:54110299)  or (SIC in (54110000:54119999) and (EmpHere<5 and EmpHere ne .)) | \| 54110200 \| Convenience stores \| \| --- \| --- \| \| 54110201 \| Convenience stores, chain \| \| 54110202 \| Convenience stores, independent \|  \| 54110000 \| Grocery stores \| \| --- \| --- \| \| 54110100 \| Supermarkets \| \| 54110101 \| Supermarkets, chain \| \| 54110102 \| Supermarkets, greater than 100,000 square feet (hypermarket) \| \| 54110103 \| Supermarkets, independent \| \| 54110104 \| Supermarkets, 55,000 - 65,000 square feet (superstore) \| \| 54110105 \| Supermarkets, 66,000 - 99,000 square feet \| \| 54110200 \| Convenience stores \| \| 54110201 \| Convenience stores, chain \| \| 54110202 \| Convenience stores, independent \| \| 54119900 \| Grocery stores, nec \| \| 54119901 \| Cooperative food stores \| \| 54119902 \| Delicatessen stores \| \| 54119903 \| Frozen food and freezer plans, except meat \| \| 54119904 \| Grocery stores, chain \| \| 54119905 \| Grocery stores, independent \| |
| **Fast food restaurant** | *National chains can be identified among 70 SIC codes for restaurants and eating places (5812) through text searches of the most recent company or trade name fields for names appearing in the Technomic Inc.’s list of the top 100 limited-service chain brands ([Technomic Inc., 2006](#_ENREF_52)).*  *Local fast food can be identified with 3 SIC codes indicating fast food restaurants and stands (58120300, 58120307, or 58120308), as well as through text searches in the most recent company or trade name fields of the 70 SIC codes for restaurants and eating places (5812).*  if SIC in (58120300, 58120307, 58120308)  OR (SIC in (58120000:58129999)  AND (INDEX(TRADENAMEHERE, "ARBYS")>0 OR INDEX(COMPANYHERE, "ARBYS") >0  OR INDEX(TRADENAMEHERE, "BASKIN ROBBINS")>0 OR INDEX(COMPANYHERE, "BASKIN ROBBINS") >0  OR INDEX(TRADENAMEHERE, "BLIMPIE")>0 OR INDEX(COMPANYHERE, "BLIMPIE") >0  OR INDEX(TRADENAMEHERE, "BOJANGLES")>0 OR INDEX(COMPANYHERE, "BOJANGLES") >0  OR INDEX(TRADENAMEHERE, "BOSTON MARKET")>0 OR INDEX(COMPANYHERE, "BOSTON MARKET") >0  OR INDEX(COMPANYHERE, "BURGER KING")>0 OR INDEX(TRADENAMEHERE,"BURGER KING")>0  OR INDEX(TRADENAMEHERE, "CHECKERS")>0 OR INDEX(COMPANYHERE, "CHECKERS") >0  OR INDEX(TRADENAMEHERE, "CHIPOTLE")>0 OR INDEX(COMPANYHERE, "CHIPOTLE") >0  OR INDEX(TRADENAMEHERE, "CHURCHS CHICKEN")>0 OR INDEX(COMPANYHERE, "CHURCHS CHICKEN") >0  OR INDEX(TRADENAMEHERE, "DAIRY QUEEN")>0 OR INDEX(COMPANYHERE, "DAIRY QUEEN") >0  OR INDEX(TRADENAMEHERE, "DOMINOS PIZZA")>0 OR INDEX(COMPANYHERE, "DOMINOS PIZZA") >0  OR INDEX(TRADENAMEHERE, "DUNKIN DONUTS")>0 OR INDEX(COMPANYHERE, "DUNKIN DONUTS") >0  OR INDEX(TRADENAMEHERE, "EL POLLO LOCO")>0 OR INDEX(COMPANYHERE, "EL POLLO LOCO") >0  OR INDEX(TRADENAMEHERE, "FUDDRUCKERS")>0 OR INDEX(COMPANYHERE, "FUDDRUCKERS") >0  OR TRADENAMEHERE="HARDEES" OR COMPANYHERE="HARDEES"  OR INDEX(TRADENAMEHERE, "JAMBA JUICE")>0 OR INDEX(COMPANYHERE, "JAMBA JUICE") >0  OR INDEX(TRADENAMEHERE, "KENTUCKY FRIED CHICKEN")>0 OR INDEX(COMPANYHERE, "KENTUCKY FRIED CHICKEN") >0  OR TRADENAMEHERE="KFC" OR COMPANYHERE="KFC"  OR INDEX(TRADENAMEHERE, "KRISPY KREME")>0 OR INDEX(COMPANYHERE, "KRISPY KREME") >0  OR INDEX(TRADENAMEHERE, "LONG JOHN SILVERS")>0 OR INDEX(COMPANYHERE, "LONG JOHN SILVERS") >0  OR INDEX(TRADENAMEHERE, "MCDONALDS")>0 OR INDEX(COMPANYHERE, "MCDONALDS") >0  OR INDEX(TRADENAMEHERE, "PANDA EXPRESS")>0 OR INDEX(COMPANYHERE, "PANDA EXPRESS") >0  OR INDEX(TRADENAMEHERE, "PAPA JOHN")>0 OR INDEX(COMPANYHERE, "PAPA JOHN") >0  OR INDEX(TRADENAMEHERE, "PIZZA HUT")>0 OR INDEX(COMPANYHERE, "PIZZA HUT") >0  OR INDEX(TRADENAMEHERE, "POPEYES")>0 OR INDEX(COMPANYHERE, "POPEYES") >0  OR INDEX(TRADENAMEHERE, "QUIZNOS")>0 OR INDEX(COMPANYHERE, "QUIZNOS") >0  OR INDEX(TRADENAMEHERE, "ROUND TABLE")>0 OR INDEX(COMPANYHERE, "ROUND TABLE") >0  OR TRADENAMEHERE="SBARRO" OR COMPANYHERE="SBARRO"  OR TRADENAMEHERE="SONIC" OR COMPANYHERE="SONIC"  OR INDEX(TRADENAMEHERE, "STARBUCKS")>0 OR INDEX(COMPANYHERE, "STARBUCKS") >0  OR TRADENAMEHERE="SUBWAY" OR COMPANYHERE="SUBWAY"  OR TRADENAMEHERE="SUBWAY SANDWICHES" OR COMPANYHERE="SUBWAY SANDWICHES"  OR TRADENAMEHERE="SUBWAY RESTAURANT" OR COMPANYHERE="SUBWAY RESTAURANT"  OR INDEX(COMPANYHERE, "TACO BELL")>0 OR INDEX(TRADENAMEHERE,"TACO BELL")>0  OR INDEX(TRADENAMEHERE, "TCBY")>0 OR INDEX(COMPANYHERE, "TCBY") >0  OR INDEX(TRADENAMEHERE, "WENDYS")>0 OR INDEX(COMPANYHERE, "WENDYS OLD") >0  OR INDEX(TRADENAMEHERE, "WHITE CASTLE")>0 OR INDEX(COMPANYHERE, "WHITE CASTLE") >0  or INDEX(COMPANYHERE, "BEN & JERRYS")>0 OR INDEX(TRADENAMEHERE, "BEN & JERRYS")>0  or INDEX(COMPANYHERE, "BEN AND JERRYS")>0 OR INDEX(TRADENAMEHERE, "BEN AND JERRYS")>0  OR INDEX(COMPANYHERE, " CARVEL ICE CREAM CAKES")>0 OR INDEX(TRADENAMEHERE," CARVEL ICE CREAM CAKES")>0  OR INDEX(COMPANYHERE, "COLD STONE CREAMERY")>0 OR INDEX(TRADENAMEHERE, "COLD STONE CREAMERY")>0  OR INDEX(COMPANYHERE, "HAAGEN-DAZS")>0 OR INDEX(TRADENAMEHERE, "HAAGEN-DAZS") >0  OR INDEX(COMPANYHERE, "HAAGENDAZS")>0 OR INDEX(TRADENAMEHERE, "HAAGENDAZS") >0  OR INDEX(COMPANYHERE, "HAAGEN DAZS")>0 OR INDEX(TRADENAMEHERE, "HAAGEN DAZS") >0  OR INDEX(COMPANYHERE, "I CANT BELIEVE ITS YOGURT")>0 OR INDEX(TRADENAMEHERE, "I CANT BELIEVE ITS YOGURT")>0  OR INDEX(COMPANYHERE, "LITTLE CAESARS PIZZA")>0 OR INDEX(TRADENAMEHERE, "LITTLE CAESARS PIZZA") >0  OR INDEX(COMPANYHERE, "SCHLOTZSKYS DELI")>0 OR INDEX(TRADENAMEHERE, "SCHLOTZSKYS DELI") >0  OR INDEX(TRADENAMEHERE,"AU BON PAIN")>0 OR INDEX(COMPANYHERE, "A B P CORPORATION")>0 OR INDEX(COMPANYHERE, "AU BON PAIN")>0 OR INDEX(TRADENAMEHERE, "A B P CORPORATION")>0  OR INDEX(TRADENAMEHERE,"AUNTIE ANNES")>0 OR INDEX(COMPANYHERE, "AUNTIE ANNES")>0  OR INDEX(TRADENAMEHERE,"CHICKEN HOLIDAY")>0 OR INDEX(COMPANYHERE, "CHICKEN HOLIDAY")>0  OR INDEX(TRADENAMEHERE,"COFFEE SHOP NORTH CENTRAL HOSP")>0 OR INDEX(COMPANYHERE, "DIRECTORS METRO FOOD SERVICE")>0  OR INDEX(TRADENAMEHERE,"COSI SANDWICH")>0 OR INDEX(COMPANYHERE, "COSI SANDWICH")>0  OR INDEX(TRADENAMEHERE, "CROWN FRIED CHICKEN")>0 OR INDEX(COMPANYHERE, "CROWN FRIED CHICKEN")>0  OR INDEX(TRADENAMEHERE,"EVERYTHING YOGURT & SALAD")>0 OR INDEX(COMPANYHERE, "EVERYTHING YOGURT & SALAD")>0  OR INDEX(TRADENAMEHERE,"GRAYS PAPAYA")>0 OR INDEX(COMPANYHERE, "GRAYS PAPAYA")>0  OR INDEX(TRADENAMEHERE, "KENNEDY FRIED CHICKEN")>0 OR INDEX(COMPANYHERE, "KENNEDY FRIED CHICKEN")>0  OR INDEX(TRADENAMEHERE, "KOSHER DELIGHT CORP")>0 OR INDEX(COMPANYHERE, "KOSHER DELIGHT CORP")>0  OR INDEX(TRADENAMEHERE,"MANHATTAN BAGEL")>0 OR INDEX(COMPANYHERE, "MANHATTAN BAGEL")>0  OR INDEX(TRADENAMEHERE,"METROPOLITAN DELI")>0 OR INDEX(COMPANYHERE, "METROPOLITAN DELI")>0  OR INDEX(TRADENAMEHERE, "MIAMI SUBS & GRILL")>0 OR INDEX(COMPANYHERE, "MIAMI SUBS CORPORATION")>0  OR INDEX(TRADENAMEHERE,"NATHANS FAMOUS")>0 OR INDEX(COMPANYHERE, "NATHANS FAMOUS")>0  OR INDEX(TRADENAMEHERE,"PAPAYA KING")>0 OR INDEX(COMPANYHERE, "PAPAYA KING")>0  OR INDEX(TRADENAMEHERE,"PUDGIES FAMOUS CHICKEN")>0 OR INDEX(COMPANYHERE, "PUDGIES FAMOUS CHICKEN")>0  OR INDEX(TRADENAMEHERE,"ROY ROGERS")>0 OR INDEX(COMPANYHERE, "ROY ROGERS")>0  OR INDEX(TRADENAMEHERE, "EL POLLO SUPREMO")>0 OR INDEX(COMPANYHERE, "SUPREME CHICKEN OF NEW JERSEY")>0  OR INDEX(TRADENAMEHERE,"TOGOS")>0 OR INDEX(COMPANYHERE, "TOGOS")>0  OR INDEX(TRADENAMEHERE,"XANDO")>0 OR INDEX(COMPANYHERE, "XANDO")>0  OR INDEX(TRADENAMEHERE,"ZORN FAMOUS CHICKEN & RIBS")>0 OR INDEX(COMPANYHERE, "ZORN FAMOUS CHICKEN & RIBS")>0  OR INDEX(TRADENAMEHERE,"ZORNS FAMOUS CHICKEN")>0 OR INDEX(COMPANYHERE, "ZORNS FAMOUS CHICKEN")>0)) | \| 58120300 \| Fast food restaurants and stands \| \| --- \| --- \| \| 58120307 \| Fast-food restaurant, chain \| \| 58120308 \| Fast-food restaurant, independent \|  \| 5812 \| Eating places \| \| --- \| --- \| |
| **Pizza restaurant** | *Includes 3 SIC codes, as well as a text search of the word “PIZZA” or “PIZZERIA” among the most recent company or trade name fields of 70 SICs for restaurants and eating places (5812). (Note: businesses already categorized as fast food restaurants are excluded)*  if (SIC in (58120600, 58120601, 58120602)  or index(CompanyHere, "PIZZA")>0  or index(TradeNameHere, "PIZZA")>0  or index(CompanyHere, "PIZZERIA")>0  or index(TradeNameHere, "PIZZERIA")>0 | \| 58120600 \| Pizza restaurants \| \| --- \| --- \| \| 58120601 \| Pizzeria, chain \| \| 58120602 \| Pizzeria, independent \|  \| 5812 \| Eating places \| \| --- \| --- \| |
| **Other restaurants – not fast food, not pizza** | *Includes 70 SIC codes (5812) if the restaurants and eating places are not already categorized as fast food or pizza.*  if SIC in (58120000:58129999) | \| 5812 \| Eating places \| \| --- \| --- \| |
| **Bakery or candy/ confectionary store** | *Includes 10 SIC codes for retail bakeries (5461), and 4 SIC codes for stores selling candy, confectionery items, or popcorn. (Note: businesses already categorized as fast food were excluded)*  if SIC in (54610000:54619999, 54419901, 54419902, 54419903, 54419905) | \| 54610000 \| Retail bakeries \| \| --- \| --- \| \| 54619900 \| Retail bakeries, nec \| \| 54619901 \| Bagels \| \| 54619902 \| Bread \| \| 54619903 \| Cakes \| \| 54619904 \| Cookies \| \| 54619905 \| Doughnuts \| \| 54619906 \| Pastries \| \| 54619907 \| Pies \| \| 54619908 \| Pretzels \| \| 54419901 \| Candy \| \| 54419902 \| Confectionery \| \| 54419903 \| Confectionery produced for direct sale on the premises \| \| 54419905 \| Popcorn, including caramel corn \| |
| **Warehouse or discount department store selling food** | *Includes a text search among the most recent company and tradename for 4 businesses.*  if INDEX(TRADENAMEHERE, "SAMS CLUB")>0 OR INDEX(COMPANYHERE, "SAMS CLUB") >0  or INDEX(TRADENAMEHERE, "COSTCO WHOLESALE")>0 OR INDEX(COMPANYHERE, "COSTCO WHOLESALE") >0  or TRADENAMEHERE="PRICE CLUB" OR COMPANYHERE="PRICE CLUB"  or INDEX(TRADENAMEHERE, "BJS WHOLESALE")>0 OR INDEX(COMPANYHERE, "BJS WHOLESALE") >0 OR COMPANYHERE="BJS" |  |
| **Multi-use physical activity venue** | *Includes 11 SIC codes for multi-use physical activity facilities such as gyms. Gyms located within community centers such as YMCAs are identified through a text search among the most recent company and tradename for 4 businesses. (Note: businesses already flagged as any of the aforementioned categories of interest were excluded)*  if SIC in (70110306, 70110307, 79910000, 79910100, 79910101, 79910102, 79910300, 79910302, 79970000, 79991127, 79999910) or  INDEX(COMPANYHERE,'JCC OF')>0 or  INDEX(COMPANYHERE,'JEWISH CMMN CTR')>0 or  INDEX(COMPANYHERE,'JEWISH CMMNTY CTR')>0 or  INDEX(COMPANYHERE,'JEWISH CMNTY CNTRE')>0 or  INDEX(COMPANYHERE,'JEWISH CMNTY CTR')>0 or  INDEX(COMPANYHERE,'JEWISH CMNTY HSE')>0 or  INDEX(COMPANYHERE,'JEWISH CMTY CENTER')>0 or  INDEX(COMPANYHERE,'JEWISH COMMNTY CTR')>0 or  INDEX(COMPANYHERE,'JEWISH COMMUNITY CENTER')>0 or  INDEX(COMPANYHERE,'JEWISH COMMUNITY CENTRE')>0 or  INDEX(COMPANYHERE,'JEWISH COMMUNITY CTR')>0 or  INDEX(COMPANYHERE,'JEWISH CMNTY CTR')>0 or  INDEX(COMPANYHERE,'JEWISH COMMUNTY CENTER')>0 or  INDEX(COMPANYHERE,'Y M & Y M H A')>0 or  INDEX(COMPANYHERE,'Y M C A')>0 or  INDEX(COMPANYHERE,'Y W C A')>0 or  INDEX(COMPANYHERE,'YM C A')>0 or  INDEX(COMPANYHERE,'YMHA')>0 or  INDEX(COMPANYHERE,'YWHA')>0 or  INDEX(COMPANYHERE,'YMCA')>0 or  INDEX(COMPANYHERE,'YMWCA')>0 or  INDEX(COMPANYHERE,'YWCA')>0 or  INDEX(COMPANYHERE,'YNG MENS & YNG WOMENS HEBREW')>0 or  INDEX(COMPANYHERE,'YOUNG MEN CHRISTIAN ASSOCIATON')>0 or  INDEX(COMPANYHERE,'YOUNG MEN YUNG WNS HBREW ASSN')>0 or  INDEX(COMPANYHERE,'YOUNG MEN YUNG WNS HEBREW ASSN')>0 or  INDEX(COMPANYHERE,'YOUNG MENS & WOMENS ASSC')>0 or  INDEX(COMPANYHERE,'YOUNG MENS & WOMENS CHRISTIA')>0 or  INDEX(COMPANYHERE,'YOUNG MENS & YOUNG WOMENS')>0 or  INDEX(COMPANYHERE,'YOUNG MENS AND YOUNG WOMENS')>0 or  INDEX(COMPANYHERE,'YOUNG MENS CHRISTIAN')>0 or  INDEX(COMPANYHERE,'YOUNG MENS CHRSTN ASSOC')>0 or  INDEX(COMPANYHERE,'YOUNG MENS HEBREW ASSOCIATION')>0 or  INDEX(COMPANYHERE,'YOUNG MENS YNG WMNS HBRW ASSN')>0 or  INDEX(COMPANYHERE,'YOUNG MENS/YOUNG WOMENS HEBREW')>0 or  INDEX(COMPANYHERE,'YOUNG MNS CHRISTN ASSN')>0 or  INDEX(COMPANYHERE,'YOUNG MNS CHRSTN ASSN')>0 or  INDEX(COMPANYHERE,'YOUNG MNS YUNG WNS HEBREW ASSN')>0 or  INDEX(COMPANYHERE,'YOUNG WNS CHRISTN ASSN')>0 or  INDEX(COMPANYHERE,'YOUNG WOMANS CHRISTIAN ASSOC')>0 or  INDEX(COMPANYHERE,'YOUNG WOMENS CHRISTIAN')>0 or  INDEX(COMPANYHERE,'YWC ASSOC')>0 or  index(TradeNameHERE,'J C C')>0 or  index(TradeNameHERE,'JCC')>0 or  index(TradeNameHERE,'JEWISH CMNTY CNTR')>0 or  index(TradeNameHERE,'JEWISH CMNTY CTR')>0 or  index(TradeNameHERE,'JEWISH COMMUNITY CENTER')>0 or  index(TradeNameHERE,'JEWISH COMMUNITY CNTR')>0 or  index(TradeNameHERE,'Y M C A')>0 or  index(TradeNameHERE,'Y W C A')>0 or  index(TradeNameHERE,'YM & YW H A')>0 or  index(TradeNameHERE,'YWHA')>0 or  index(TradeNameHERE,'YMCA')>0 or  index(TradeNameHERE,'YMWCA')>0 or  index(TradeNameHERE,'YOUNG MENS CHRISTIAN ASSOCIAT')>0 or  index(TradeNameHERE,'YOUNG MNS YUNG WNS HEBREW ASSN')>0 or  index(TradeNameHERE,'YOUNG WOMENS CHRISTIAN ASSN')>0 or  index(TradeNameHERE,'YOUNG WOMENS CHRISTIAN ASSOC')>0 or  index(TradeNameHERE,'YWCA')>0 | \| 70110306 \| YMCA/YMHA hotel \| \| --- \| --- \| \| 70110307 \| YWCA/YWHA hotel \| \| 79910000 \| Physical fitness facilities \| \| 79910100 \| Physical fitness clubs with training equipment \| \| 79910101 \| Athletic club and gymnasiums, membership \| \| 79910102 \| Health club \| \| 79910300 \| Exercise facilities \| \| 79910302 \| Exercise salon \| \| 79970000 \| Membership sports and recreation clubs \| \| 79991127 \| Physical fitness instruction \| \| 79999910 \| Recreation center \| |
| **Light/moderate physical activity venue** | *Includes 55 SIC codes. (Note: businesses already flagged as a multi-use physical activity venue are excluded)*  if SIC in (39490103, 70320301, 79110000, 79110100, 79110101, 79110200, 79110201, 79110202, 79110203, 79110204, 79330000, 79339903, 79920000, 79970101, 79970200, 79970201, 79970202, 79970204, 79970300, 79970301, 79970302, 79979904, 79979906, 79979908, 79990200, 79990202, 79990203, 79990204, 79990205, 79990601, 79990700, 79990701, 79990702, 79990703, 79990704, 79990705, 79991102, 79991104, 79991109, 79991115, 79991121, 79991123, 79991200, 79991201, 79991202, 79991205, 79991400, 79991402, 79991409, 79991411, 79991512, 79991601, 79991602, 79999903, 82999903) | \| 39490103 \| Driving ranges, golf, electronic \| \| --- \| --- \| \| 70320301 \| Fishing camp \| \| 79110000 \| Dance studios, schools, and halls \| \| 79110100 \| Dance hall services \| \| 79110101 \| Dance hall or ballroom operation \| \| 79110200 \| Dance instructor and school services \| \| 79110201 \| Childrens' dancing school \| \| 79110202 \| Dance instructor \| \| 79110203 \| Dance studio and school \| \| 79110204 \| Professional dancing school \| \| 79330000 \| Bowling centers \| \| 79339903 \| Ten pin center \| \| 79920000 \| Public golf courses \| \| 79970101 \| Curling club, membership \| \| 79970200 \| Boating and swimming clubs \| \| 79970201 \| Beach club, membership \| \| 79970202 \| Boating club, membership \| \| 79970204 \| Yacht club, membership \| \| 79970300 \| Gun and hunting clubs \| \| 79970301 \| Gun club, membership \| \| 79970302 \| Hunting club, membership \| \| 79979904 \| Country club, membership \| \| 79979906 \| Golf club, membership \| \| 79979908 \| Riding club, membership \| \| 79990200 \| Golf services and professionals \| \| 79990202 \| Golf driving range \| \| 79990203 \| Golf professionals \| \| 79990204 \| Golf, pitch-n-putt \| \| 79990205 \| Miniature golf course operation \| \| 79990601 \| Curling rinks \| \| 79990700 \| Shooting facilities and archery lanes \| \| 79990701 \| Archery lanes \| \| 79990702 \| Shooting gallery \| \| 79990703 \| Shooting range operation \| \| 79990704 \| Skeet shooting facility \| \| 79990705 \| Trapshooting facility, non-membership \| \| 79991102 \| Baseball instruction school \| \| 79991104 \| Bowling instruction \| \| 79991109 \| Gymnastic instruction, non-membership \| \| 79991115 \| Sailing instruction \| \| 79991121 \| Surfing instruction \| \| 79991123 \| Yoga instruction \| \| 79991200 \| Riding and rodeo services \| \| 79991201 \| Riding academy and school \| \| 79991202 \| Riding stable \| \| 79991205 \| Saddlehorse rental \| \| 79991400 \| Beach and water sports equipment rental and services \| \| 79991402 \| Bathing beach, non-membership \| \| 79991409 \| Rowboat and canoe rental \| \| 79991411 \| Surfing equipment rental \| \| 79991512 \| Waterslide operation \| \| 79991601 \| Hunting guides \| \| 79991602 \| Rafting tours \| \| 79999903 \| Baseball batting cage \| \| 82999903 \| Baton instruction \| |
| **Vigorous physical activity venue** | *Includes 37 SIC codes. (Note: businesses already flagged as multi-use physical activity venues are excluded)*  if SIC in (70110201, 79410104, 79410201, 79910301, 79970100, 79970102, 79970203, 79970402, 79970403, 79970500, 79970502, 79970503, 79970504, 79990100, 79990101, 79990102, 79990300, 79990301, 79990302, 79990303, 79990501, 79990600, 79990602, 79990603, 79991103, 79991107, 79991110, 79991111, 79991112, 79991113, 79991116, 79991118, 79991119, 79991120, 79991122, 79991412, 82999914))) | \| 70110201 \| Ski lodge \| \| --- \| --- \| \| 79410104 \| Ice hockey club \| \| 79410201 \| Boxing and wrestling arena \| \| 79910301 \| Aerobic dance and exercise classes \| \| 79970100 \| Ice sports \| \| 79970102 \| Hockey club, except professional and semi- professional \| \| 79970203 \| Swimming club, membership \| \| 79970402 \| Football club, except professional and semi- professional \| \| 79970403 \| Polo club, membership \| \| 79970500 \| Indoor/outdoor court clubs \| \| 79970502 \| Racquetball club, membership \| \| 79970503 \| Squash club, membership \| \| 79970504 \| Tennis club, membership \| \| 79990100 \| Tennis services and professionals \| \| 79990101 \| Tennis club, non-membership \| \| 79990102 \| Tennis courts, outdoor/indoor: non-membership \| \| 79990300 \| Indoor court clubs \| \| 79990301 \| Handball courts, non-membership \| \| 79990302 \| Racquetball club, non-membership \| \| 79990303 \| Squash club, non-membership \| \| 79990501 \| Bicycle rental \| \| 79990600 \| Skating rink operation services \| \| 79990602 \| Ice skating rink operation \| \| 79990603 \| Roller skating rink operation \| \| 79991103 \| Basketball instruction school \| \| 79991107 \| Diving instruction, underwater \| \| 79991110 \| Hockey instruction school \| \| 79991110 \| Hockey instruction school \| \| 79991111 \| Judo instruction \| \| 79991112 \| Karate instruction \| \| 79991113 \| Martial arts school, nec \| \| 79991116 \| Scuba and skin diving instruction \| \| 79991118 \| Skating instruction, ice or roller \| \| 79991119 \| Ski instruction \| \| 79991120 \| Sports instruction, schools and camps \| \| 79991122 \| Swimming instruction \| \| 79991412 \| Swimming pool, non-membership \| \| 82999914 \| Self-defense and athletic instruction \| |
| **Urgent care and hospital facilities** | *Includes 91 SIC codes.*  if SIC in (80110200, 80110201, 80110204, 80620000:80629999, 80690000, 80690200, 80690201, 80690300, 80690301, 80699901, 80699902, 80699903, 80699904, 80699905) | \| 80110200 \| Medical centers \| \| --- \| --- \| \| 80110201 \| Ambulatory surgical center \| \| 80110204 \| Freestanding emergency medical center \| \| 80620000 \| General medical and surgical hospitals \| \| 80629900 \| General medical and surgical hospitals, nec \| \| 80629901 \| Hospital, affiliated with AMA residency \| \| 80629902 \| Hospital, med school affiliated with nursing and residency \| \| 80629903 \| Hospital, medical school affiliated with residency \| \| 80629904 \| Hospital, medical school affiliation \| \| 80629905 \| Hospital, professional nursing school \| \| 80629906 \| Hospital, professional nursing school with AMA residency \| \| 80629907 \| Hospital, AMA approved residency \| \| 80690000 \| Specialty hospitals, except psychiatric \| \| 80690200 \| Chronic disease hospital \| \| 80690201 \| Cancer hospital \| \| 80690300 \| Respiratory hospital \| \| 80690301 \| Tuberculosis hospital \| \| 80699901 \| Childrens' hospital \| \| 80699902 \| Eye, ear, nose, and throat hospital \| \| 80699903 \| Geriatric hospital \| \| 80699904 \| Maternity hospital \| \| 80699905 \| Orthopedic hospital \| |
| **Office or clinic of health practitioner** | *Includes 22 SIC codes.*  if SIC in (80110000: 80110110, 80110202, 80110205, 80110500: 80119905, 80310000:80490201, 80499900, 80499902, 80499903, 80499904, 80499906, 80499908, 80499909, 80920000, 80930000, 80930200:80939901, 80939903, 80939905, 80990103, 80990104, 80990200, 80990201, 80990203, 80999905, 80999906, 80999907) | \| 80110000 \| Offices and clinics of medical doctors \| \| --- \| --- \| \| 80110100 \| Internal medicine practitioners \| \| 80110101 \| Cardiologist and cardio-vascular specialist \| \| 80110102 \| Endocrinologist \| \| 80110103 \| Gastronomist \| \| 80110104 \| Hematologist \| \| 80110105 \| Internal medicine, physician/surgeon \| \| 80110106 \| Nephrologist \| \| 80110107 \| Neurologist \| \| 80110108 \| Oncologist \| \| 80110109 \| Pulmonary specialist, physician/surgeon \| \| 80110110 \| Neurosurgeon \| \| 80110202 \| Clinic, operated by physicians \| \| 80110205 \| Primary care medical clinic \| \| 80110500 \| Specialized medical practitioners, except internal \| \| 80110501 \| Allergist \| \| 80110502 \| Anesthesiologist \| \| 80110503 \| Dermatologist \| \| 80110504 \| Diabetes specialist, physician/surgeon \| \| 80110505 \| Ears, nose, and throat specialist: physician/surgeon \| \| 80110506 \| Eyes, ears, nose, and throat specialist: physician/surgeon \| \| 80110507 \| Geriatric specialist, physician/surgeon \| \| 80110508 \| Gynecologist \| \| 80110509 \| Immunologist \| \| 80110510 \| Infectious disease specialist, physician/surgeon \| \| 80110511 \| Obstetrician \| \| 80110512 \| Oculist \| \| 80110513 \| Opthalmologist \| \| 80110514 \| Orthopedic physician \| \| 80110515 \| Pathologist \| \| 80110516 \| Pediatrician \| \| 80110517 \| Plastic surgeon \| \| 80110518 \| Proctologist \| \| 80110519 \| Radiologist \| \| 80110520 \| Rheumatology specialist, physician/surgeon \| \| 80110521 \| Sports medicine specialist, physician \| \| 80110522 \| Thoracic physician \| \| 80110523 \| Urologist \| \| 80110524 \| Fertility specialist, physician \| \| 80119900 \| Offices and clinics of medical doctors, nec \| \| 80119901 \| General and family practice, physician/surgeon \| \| 80119902 \| Occupational and industrial specialist, physician/surgeon \| \| 80119903 \| Physical medicine, physician/surgeon \| \| 80119904 \| Physicians' office, including specialists \| \| 80119905 \| Surgeon \| \| 80310000 \| Offices and clinics of osteopathic physicians \| \| 80410000 \| Offices and clinics of chiropractors \| \| 80420000 \| Offices and clinics of optometrists \| \| 80420100 \| Specialized optometrists \| \| 80420101 \| Contact lense specialist optometrist \| \| 80420102 \| Geriatric specialist optometrist \| \| 80420103 \| Low vision specialist optometrist \| \| 80420104 \| Pediatric specialist optometrist \| \| 80420105 \| Visual training specialist optometrist \| \| 80429900 \| Offices and clinics of optometrists, nec \| \| 80429901 \| Group and corporate practice, optometrist \| \| 80430000 \| Offices and clinics of podiatrists \| \| 80490000 \| Offices of health practitioner \| \| 80490100 \| Nutrition specialist \| \| 80490101 \| Dietician \| \| 80490102 \| Nutritionist \| \| 80490200 \| Physical therapist \| \| 80490201 \| Physiotherapist \| \| 80499900 \| Offices of health practitioners, nec \| \| 80499902 \| Audiologist \| \| 80499903 \| Biofeedback therapist \| \| 80499904 \| Chiropodist \| \| 80499906 \| Inhalation therapist \| \| 80499908 \| Naturopath \| \| 80499909 \| Occupational therapist \| \| 80920000 \| Kidney dialysis centers \| \| 80930000 \| Specialty outpatient clinics, nec \| \| 80930200 \| Respiratory health clinic \| \| 80930201 \| Respiratory therapy clinic \| \| 80930202 \| Smoking clinic \| \| 80930300 \| Family planning and birth control clinics \| \| 80930301 \| Abortion clinic \| \| 80930302 \| Birth control clinic \| \| 80930303 \| Family planning clinic \| \| 80939900 \| Specialty outpatient clinics, nec, nec \| \| 80939901 \| Biofeedback center \| \| 80939903 \| Rehabilitation center, outpatient treatment \| \| 80939905 \| Weight loss clinic, with medical staff \| \| 80990103 \| Blood pressure testing \| \| 80990104 \| Plasmapherous center \| \| 80990200 \| Physical examination and testing services \| \| 80990201 \| Health screening service \| \| 80990203 \| Physical examination service, insurance \| \| 80999905 \| Medical services organization \| \| 80999906 \| Nutrition services \| \| 80999907 \| Osteoporosis center \| |
| **Residential facility with health care (e.g., nursing home)** | *Includes 21 SIC codes.*  if SIC in (80510000, 80519900, 80519901, 80519902, 80520000, 80529900, 80529902, 80590000, 80599901, 80599904, 80599905, 80599906, 83610000, 83610300, 83610400:83610499, 83619900, 83619901, 83619904) | \| 80510000 \| Skilled nursing care facilities \| \| --- \| --- \| \| 80519900 \| Skilled nursing care facilities, nec \| \| 80519901 \| Convalescent home with continuous nursing care \| \| 80519902 \| Extended care facility \| \| 80520000 \| Intermediate care facilities \| \| 80529900 \| Intermediate care facilities, nec \| \| 80529902 \| Personal care facility \| \| 80590000 \| Nursing and personal care, nec \| \| 80599901 \| Convalescent home \| \| 80599904 \| Nursing home, except skilled and intermediate care facility \| \| 80599905 \| Personal care home, with health care \| \| 80599906 \| Rest home, with health care \| \| 83610000 \| Residential care \| \| 83610300 \| Residential care for the handicapped \| \| 83610400 \| Geriatric residential care \| \| 83610401 \| Aged home \| \| 83610402 \| Old soldiers' home \| \| 83610403 \| Rest home, with health care incidental \| \| 83619900 \| Residential care, nec \| \| 83619901 \| Destitute home \| \| 83619904 \| Rehabilitation center, residential: health care incidental \| |
| **Pharmacies** | *Includes 8 SIC codes.*  if SIC in (51220000, 51220300, 51229900, 59120000:59129902, 80110203) | \| 51220000 \| Drugs, proprietaries, and sundries \| \| --- \| --- \| \| 51220300 \| Drugs and drug proprietaries \| \| 51229900 \| Drugs, proprietaries, and sundries, nec \| \| 59120000 \| Drug stores and proprietary stores \| \| 59129900 \| Drug stores and proprietary stores, nec \| \| 59129901 \| Drug stores \| \| 59129902 \| Proprietary (non-prescription medicine) stores \| \| 80110203 \| Dispensery, operated by physicians \| |
| **Mental health care** | *Includes 28 SIC codes.*  if SIC in (80110400, 80110401, 80110402, 80110403, 80490400, 80490401, 80490402, 80490403, 80490404, 80519903, 80529901, 80599903, 80630000, 80639900, 80639901, 80690100, 80690101, 80690102, 80930100, 80930101, 80930102, 80930103, 80939902, 83610302, 83610304, 83619902, 83619903, 83619905) | \| 80110400 \| Psychiatrists and psychoanalysts \| \| --- \| --- \| \| 80110401 \| Psychiatric clinic \| \| 80110402 \| Psychiatrist \| \| 80110403 \| Psychoanalyst \| \| 80490400 \| Psychologist, psychotherapist and hypnotist \| \| 80490401 \| Clinical psychologist \| \| 80490402 \| Hypnotist \| \| 80490403 \| Psychiatric social worker \| \| 80490404 \| Psychotherapist, except M.D. \| \| 80519903 \| Mental retardation hospital \| \| 80529901 \| Home for the mentally retarded, with health care \| \| 80599903 \| Home for the mentally retarded, ex. skilled or intermediate \| \| 80630000 \| Psychiatric hospitals \| \| 80639900 \| Psychiatric hospitals, nec \| \| 80639901 \| Mental hospital, except for the mentally retarded \| \| 80690100 \| Substance abuse hospitals \| \| 80690101 \| Alcoholism rehabilitation hospital \| \| 80690102 \| Drug addiction rehabilitation hospital \| \| 80930100 \| Substance abuse clinics (outpatient) \| \| 80930101 \| Alcohol clinic, outpatient \| \| 80930102 \| Detoxification center, outpatient \| \| 80930103 \| Drug clinic, outpatient \| \| 80939902 \| Mental health clinic, outpatient \| \| 83610302 \| Mentally handicapped home \| \| 83610304 \| Retarded home \| \| 83619902 \| Emotionally disturbed home \| \| 83619903 \| Halfway group home, persons with social or personal problems \| \| 83619905 \| Self-help group home \| |
| **Dental care** | *Includes 16 SIC codes.*  if SIC in (80210000, 80210100, 80210101, 80210102, 80210103, 80210104, 80210105, 80210106, 80210107, 80210108, 80210200, 80210201, 80210202, 80219901, 80219902, 80490501) | \| 80210000 \| Offices and clinics of dentists \| \| --- \| --- \| \| 80210100 \| Specialized dental practitioners \| \| 80210101 \| Dental surgeon \| \| 80210102 \| Endodontist \| \| 80210103 \| Maxillofacial specialist \| \| 80210104 \| Oral pathologist \| \| 80210105 \| Orthodontist \| \| 80210106 \| Pedodontist \| \| 80210107 \| Periodontist \| \| 80210108 \| Prosthodontist \| \| 80210200 \| Dental clinics and offices \| \| 80210201 \| Dental clinic \| \| 80210202 \| Dentists' office \| \| 80219901 \| Dental insurance plan \| \| 80219902 \| Group and corporate practice, dentist \| \| 80490501 \| Dental hygienist \| |
| **Bank** | *Includes 15 SIC codes.*  if SIC in (60210000, 60219900, 60219901, 60220000, 60229900, 60229901, 60290000, 60350000, 60359900, 60359901, 60359902, 60360000, 60369900, 60369901, 60369902) | \| 60210000 \| National commercial banks \| \| --- \| --- \| \| 60219900 \| National commercial banks, nec \| \| 60219901 \| National trust companies with deposits, commercial \| \| 60220000 \| State commercial banks \| \| 60229900 \| State commercial banks, nec \| \| 60229901 \| State trust companies accepting deposits, commercial \| \| 60290000 \| Commercial banks, nec \| \| 60350000 \| Federal savings institutions \| \| 60359900 \| Federal savings institutions, nec \| \| 60359901 \| Federal savings and loan associations \| \| 60359902 \| Federal savings banks \| \| 60360000 \| Savings institutions, except federal \| \| 60369900 \| Savings institutions, except federal, nec \| \| 60369901 \| Savings and loan associations, not federally chartered \| \| 60369902 \| State savings banks, not federally chartered \| |
| **Credit Union** | *Includes 4 SIC codes.*  if SIC in (60610000,60620000,60629900,60629901) | \| 60610000 \| Federal credit unions \| \| --- \| --- \| \| 60620000 \| State credit unions \| \| 60629900 \| State credit unions, nec \| \| 60629901 \| State credit unions, not federally chartered \| |
| **Potential destination** | *Includes SIC codes 53 (retail), 54 (food stores), 58 (eating and drinking places), 59 (miscellaneous retail), 78 (motion pictures), 83 (social services), and 84 (museums, art galleries & gardens), excluding those with 250+ employees in the most recent year. (Note: businesses already flagged in one of the aforementioned 24 categories are excluded)*  if (SIC in (53000000:53999999) and (EmpHere<250 and EmpHere ne .))  or (SIC in (54000000:54999999) and (EmpHere<250 and EmpHere ne .))  or (SIC in (58000000:58999999) and (EmpHere<250 and EmpHere ne .))  or (SIC in (59000000:59999999) and (EmpHere<250 and EmpHere ne .))  or (SIC in (78000000:78999999) and (EmpHere<250 and EmpHere ne .))  or (SIC in (83000000:83999999) and (EmpHere<250 and EmpHere ne .))  or (SIC in (84000000:84999999) and (EmpHere<250 and EmpHere ne .)) | \| 53 \| General Merchandise Stores \| \| --- \| --- \| \| 54 \| Food Stores \| \| 58 \| Eating and Drinking Places \| \| 59 \| Miscellaneous Retail \| \| 78 \| Motion Pictures \| \| 83 \| Social Services \| \| 84 \| Museums, Art Galleries and Botanical and Zoological Gardens \| |
