# Supplementary material for: Measuring health-relevant businesses over 21 years: refining the National Establishment Time-Series (NETS), a dynamic longitudinal data set
Source: BMC Res Notes. 2015 Sep 29;8:507. doi: 10.1186/s13104-015-1482-4 (PMC4588464; doi:10.1186/s13104-015-1482-4)
Supplement: Supplementary file 4 — 10.1186/s13104-015-1482-4 Information in greater detail on our geocoding processes. [file 13104_2015_1482_MOESM4_ESM.docx]

The authors advise investing in a faster computer to work with “big data” to avoid frustration as there is non-trivial run-time for commands in statistical programs and geographical information systems (GIS) on a standard desktop computer.

Geographical code processes were developed in Python 2.7 (arcpy, time, datetime, csv, sys, traceback). Due to file size limitations for certain formats, as well as software file format limitations, only a few specific file formats were used during the multi-stepped data-cleaning and geoprocessing methods. In ArcGIS the preferred file format is the ESRI File Geodatabase (which has very large table and feature class size limitations of 1 terabyte as well as 64 character field name length and allows for over 4 billion rows and 65,000+ fields). Stata files were used for simple field calculations as well as relational merges (joins). Stata performs much faster and reliable merges (joins) than ArcGIS. The preferred file format for moving data in and out of ArcGIS is the comma-separated values file format (.csv). The .csv file format is ideal for two reasons; it allows for more characters in field names than the .dbf (an older and ubiquitous table file format in GIS software) and it has no limit in file size or fields or number of rows. Also, it may be opened in text-editing software or saved as an Excel file and imported directly into Stata.

Once adequate geocoding and table related code is prepared the computer processing takes about 1.5-2 weeks to run in a standard 40 hour workweek. The amount of personnel time was reduced by automating much of the processing using code, but is still broken down into steps where the code must be initiated. It may be possible, with significant code development and testing time, to write a program to automate the entire process to run without human intervention. One way to do this would be to initiate all the code sequentially using Windows Task Scheduler or command line tools. Another method would involve writing and running everything using Python, thus making heavy use of the table tools for the .csv Python module.

The local NYC geocoder, Geosupport, was released in late September 2013 as an application program interface (API) (Geoclient API – Beta) using standard web requests that returns XML or JSON files. Running Geosupport desktop for the approximately 6 million primary and secondary addresses takes about 4 days of computer processing time. Supposedly this API can handle a large number of requests. If this process were run again it would be worthwhile to consider using this API rather than Geosupport Desktop, as it may be possible to geocode these results much faster. One important point to note - when using the Geoclient API, the requests and returns and being submitted over the web so sensitive study subject data should not be sent to the Geoclient API.

For each of our three geocoders, the coordinates corresponding to the primary address were prioritized over the secondary address because the coordinates provided by NETS for the secondary address were more frequently missing (90% missing), therein limiting comparisons. Of our final prioritized address coordinates, 37.5% of business locations were geocoded by Geosupport, 51.5% by ESRI 2010 Business Analyst to the point level, and 9.7% to the street segment. Business Analyst point level and street segment results were considered valid only if the returned geocoded address coordinates were accompanied by a matched address. Business Analyst matching options were set at 70-10-70 for spelling sensitivity, minimum candidate score, and minimum match score. Both the primary and secondary addresses of 37,670 (1.3%) business locations failed to geocode with any of our three geocoders. For these locations we used the primary address coordinates provided in NETS. In total, 98.4% of our final prioritized coordinates were from a primary address.

The more spatially precise address coordinates in NETS (block face or street segment accuracy), were within 100 meters of the coordinates returned by our three geocoders 78% to 94% of the time, depending on the geocoder. For a given address, the coordinates returned by our three geocoders were more proximal to one another, than to the corresponding coordinate in NETS. Where two addresses for a given business location were re-geocoded by Business Analyst to the point level (n=1,856,378), 95% were within 100 meters of one another.

The proximity of the coordinates returned by our three geocoders to one another for a given address gave us confidence in the accuracy of our GIS results. The large proportion of primary and secondary addresses that geocoded within 100 meters of one another substantiated our notion that they generally represent the same location. Discordance between the primary and secondary address may occur due to error, if the same address is recorded with a different character string across datasets, or if there was a minor move within the same zip code since the move dataset tracks only moves in which both the five-digit zip code and street address change between years.
